# Methionine Mutations of Outer Membrane Protein X Influence Structural Stability and Beta-Barrel Unfolding

**DOI:** 10.1371/journal.pone.0079351

**Published:** 2013-11-12

**Authors:** Deepti Chaturvedi, Radhakrishnan Mahalakshmi

**Affiliations:** Molecular Biophysics Laboratory, Department of Biological Sciences, Indian Institute of Science Education and Research, Bhopal, Madhya Pradesh, India; Aligarh Muslim University, India

## Abstract

We report the biochemical and biophysical characterization of outer membrane protein X (OmpX), an eight-stranded transmembrane β-barrel from *E. coli*, and compare the barrel behavior with a mutant devoid of methionine residues. Transmembrane outer membrane proteins of bacterial origin are known to display high tolerance to sequence rearrangements and mutations. Our studies with the triple mutant of OmpX that is devoid of all internal methionine residues (M18L; M21L; M118L) indicate that Met replacement has no influence on the refolding efficiency and structural characteristics of the protein. Surprisingly, the conserved substitution of Met→Leu leads to barrel destabilization and causes a lowering of the unfolding free energy by a factor of ∼8.5 kJ/mol, despite the mutations occurring at the loop regions. We report that the barrel destabilization is accompanied by a loss in cooperativity of unfolding in the presence of chemical denaturants. Furthermore, we are able to detect an unfolding intermediate in the Met-less barrel, whereas the parent protein exhibits a classic two-state unfolding. Thermal denaturation measurements also suggest a greater susceptibility of the OmpX barrel to heat, in the Met-less construct. Our studies reveal that even subtle variations in the extra-membrane region of rigid barrel structures such as OmpX, may bear severe implications on barrel stability. We propose that methionines contribute to efficient barrel structuring and protein-lipid interactions, and are therefore important elements of OmpX stability.

## Introduction

Membrane proteins constitute over 30% of the cellular proteome, and have long been recognized as indispensable entities of the cell. They are implicated in a wide number of fatal and presently incurable diseases, including Alzheimer’s, Parkinson’s, stroke and cancer [1]–[3]. Despite their biological importance, surprisingly little is currently known about their folding and stability. For soluble proteins, folding pathway(s) have been mapped extensively using a combination of mutational analyses, *in silico* experiments as well as structure determination [4]–[7]. However, in the case of membrane proteins, our current knowledge of their physico-chemical properties is based largely on the behavior of a select set of well-characterized representatives of the α-helical family and β-barrel category, such as bacteriorhodopsin, *Escherichia coli* OmpA and PagP [8]–[14].

Outer membrane protein X (OmpX) is one of the structurally characterized members of the β-barrel family of bacterial transmembrane proteins [15]. OmpX is believed to play a role in neutralization of host defense and complement binding, thereby conferring pathogenicity to *E. coli* [16]–[19]. The structure of the H100N mutant of OmpX has been determined using X-ray diffraction [18], solution NMR methods in micelles [20] and solid state NMR studies in lipid bilayers and bicelles [19], [21]. Similar to OmpA and PagP (other β-barrels from *E. coli*), OmpX forms an asymmetric 8-stranded barrel with short loop segments facing the inter-membrane space and longer loop regions that is in contact with the external environment.

OmpX folds readily in various lipids and detergents *in vitro*, and the folded protein exhibits a surprisingly high thermostability in lipid micelles, bilayers and nanodiscs, despite being of mesophilic origin [14]. Duplication and strand shuffling have been attempted before in this protein [22], and research on such sequences has shown that most of the chimeric constructs undergo complete refolding [22]. These studies suggest that OmpX is highly tolerant to sequence rearrangements, strand shuffling and mutations. Indeed, the stability of OmpX has attracted its use in phage display libraries [23].

We specifically address the effect of methionine mutations on the structure and stability of OmpX H100N (henceforth referred to as OmpX^HN^), in this study. In proteins, the methionine side chain is particularly susceptible to oxidative modifications. Mutation of residues such as cysteine and methionine, for *in vitro* experiments, is carried out in proteins where prolonged sample stability is vital [24], [25], for instance, in crystallization studies. Conserved substitution of these amino acids, to Leu (in the place of Met) or Ala/Ser (for Cys) are carried out, assuming that such conserved substitutions do not affect the structure or biophysical properties of the protein [26]. Furthermore, enzymatic assays in such mutants demonstrate comparable activity in both native and mutant proteins. Methionine remains one of the least understood amino acids, despite several diseases associated with this residue, including Alzheimer’s [27]. Methionine is particularly susceptible to irreversible oxidative modification by reactive oxygen species [28], [29], which impairs crystallization and affects enzymatic activity. Met oxidation also interferes in cyanogen bromide mediated protein cleavage chemistries, leading to undesirable by-products. Owing to its low occurrence in proteins, methionine is often replaced by leucine, without major effect on the protein structure [25], [30]–[37]. Hence, we addressed whether the conserved substitution of a Met residue to leucine would be well-accommodated and tolerated in a membrane protein with known stability, such as OmpX. Furthermore, we have probed whether there are biophysical implications of such conserved substitutions on the folding/unfolding pathways of membrane proteins. Besides, mutation of internal Met residues would also facilitate the use of such mutants as fusion constructs for protein over-expression and subsequent cyanogen bromide cleavage [38].

OmpX has three methionine residues, at positions 18, 21 and 118. All of these can be mapped to the extra-membrane segment, with two (18 and 21) facing the extracellular region and one (118) situated in the periplasm. All three methionine residues are therefore solvent exposed and are amenable to oxidation. Unlike their soluble counterparts, multi-pass membrane proteins such as transmembrane barrels, show three distinct zones structurally [39]. The hydrophobic core, which is enveloped by the lipid chains, is essential to maintain structural stability. 8-stranded barrels in *E. coli* exhibit a high degree of sequence conservation in this region. The membrane anchoring region forms the girdle and is rich in aromatic residues which serve as the ‘stop transfer’ signal during protein synthesis and folding [40]. This girdle forms the interface between the hydrophobic lipid core, solvent and polar lipid headgroups. This region also determines protein stability and may contribute to folding rates. The region with greatest variability and increased mutability is the loop region. Loop segments vary in length and residue composition across the various OMPs of *E. coli* and other Gram negative bacteria. OmpX is known to demonstrate high tolerance to mutations in the loop regions [23]. Since Met residues of OmpX are present in the loop segments, we anticipated a Met→Leu mutation to have no effect on barrel stability.

**Figure 1:**
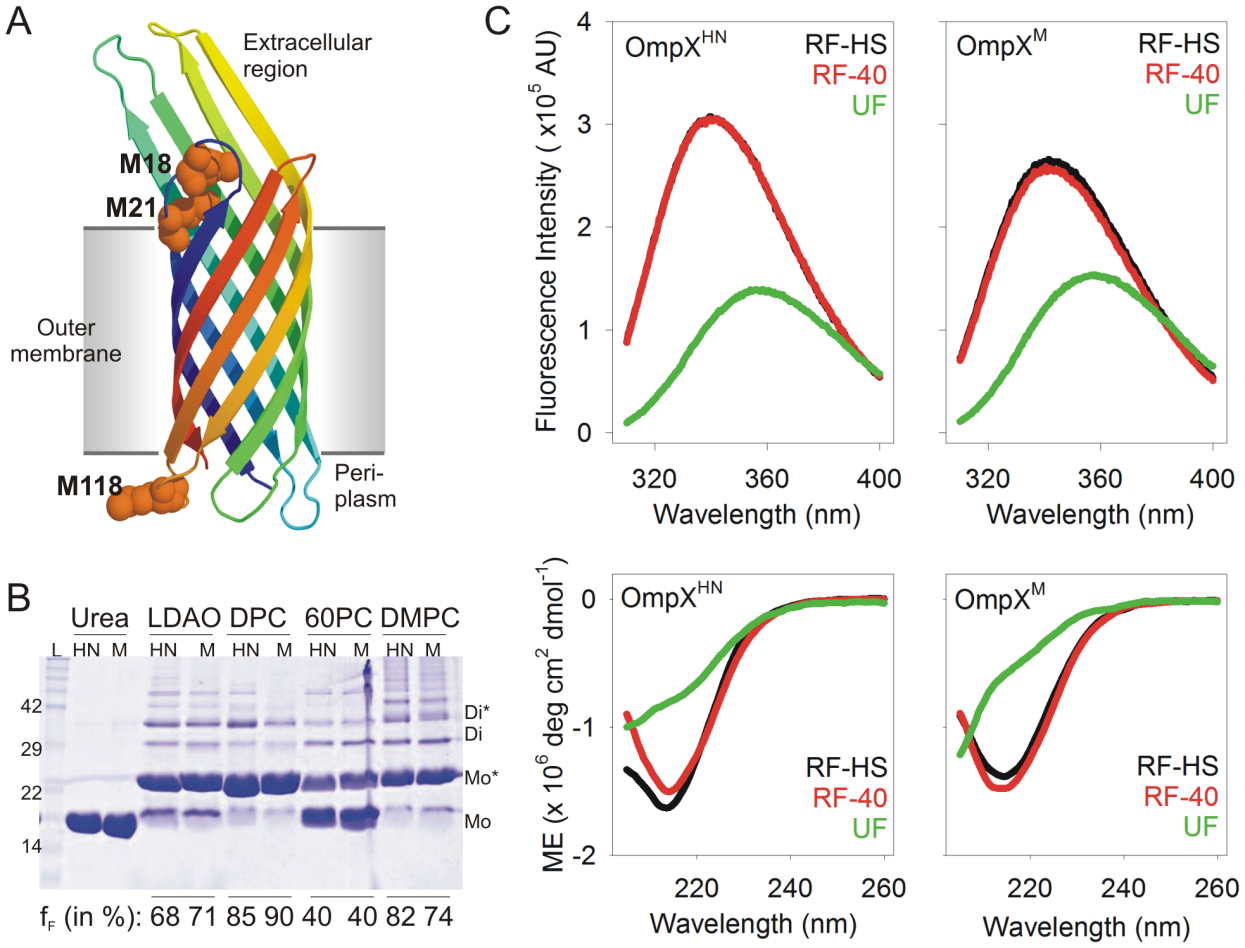
OmpX^HN^ and OmpX^M^ show comparable refolding efficiency. (A) Cartoon representation of OmpX^HN^ (PDB ID: 1QJ8) generated using PyMol [62], with the three Met residues at positions 18, 21 and 118, rendered as spheres. These methionines have been mutated to leucine in OmpX^M^. (B) SDS-PAGE analysis of unboiled samples, comparing the refolding efficiency of OmpX^HN^ (labeled HN) and OmpX^M^ (labeled M). Refolding of these samples was achieved by the slow folding method at 40°C, in lipids and detergents mentioned above each lane. Upon folding, OmpX migrates at ∼22 kDa, compared to the unfolded protein (labeled urea) at ∼16 kDa. Fraction folded (f_F_), determined by densitometry using band intensity of the monomeric species, is indicated below each lane. Samples were not centrifuged to remove any precipitated protein, so that a correct estimate of the folding efficiency in each condition could be obtained. Note the formation of higher order oligomers for both proteins, upon refolding, as observed earlier for OmpA [63]. LDAO: lauryldimethylamine oxide; DPC: n-dodecylphosphocholine; 60PC: 6∶0 diether PC; DMPC: 1,2 dimyristoyl-sn-glycero-3-phosphocholine; L: protein MW marker; Mo: unfolded monomer; Mo*: folded monomer; Di: unfolded dimer; Di*: folded dimer. (C) Fluorescence emission (top panels) and far-UV CD spectra (bottom panels) of OmpX^HN^ (left) and OmpX^M^ (right), refolded using heat shock (RF-HS) and slow folding at 40°C (RF-40). Spectra of unfolded protein samples (UF) in 8 M GdmHCl or directly re-suspended in buffer, for fluorescence and CD scans, respectively, are also provided for comparison. The labels reflect color codes used for the respective samples. Note that in the top left panel, the fluorescence emission spectrum of RF-HS OmpX^HN^ (black curve) is directly underneath the spectrum of RF-40 OmpX^HN^ (red curve).

Here, we have investigated the thermodynamic properties of OmpX^HN^, and compared the results with the Met→Leu mutant OmpX^M^ where all three Met residues (at 18, 21 and 118) in OmpX^HN^ were replaced with Leu (see Figure 1A). We have examined the response of both proteins, refolded in the detergent lauryldimethylamine oxide (LDAO), to reversible chemical and thermal denaturation. Our experiments reveal that Met mutations dramatically affect the unfolding free energy (ΔG^0^_U_) of the protein. Using anisotropy measurements, we observe the presence of an unfolding intermediate for the mutant protein OmpX^M^, while the parent protein OmpX^HN^ follows the classic two-state unfolding pathway. Our results indicate that structural stabilization is perturbed upon Met mutation in OmpX. The implications of our observations on OmpX stability, and the role of methionines in barrel structure, have been discussed.

## Materials and Methods

### Mutant Generation

Wild type OmpX gene (without the signal sequence) was a kind gift from Prof. Kurt Wüthrich at the Scripps Research Institute, CA. OmpX H100N, the OmpX version that has been structurally examined [18]–[20], was generated as reported earlier [14] and is referred to in this study as OmpX^HN^. OmpX^HN^ has three internal Met residues, located at positions 18, 21 and 118. These methionines were mutated to leucine residues using reported protocols [41] for site directed mutagenesis (SDM), as described earlier [38]. The resulting Met-less construct is referred to as OmpX^M^.

### Protein Refolding

Both OmpX^HN^ and OmpX^M^ were over-produced as inclusion bodies in *E. coli* C41 cells grown in Luria-Bertini medium or M9 medium using ^15^N ammonium sulfate as the sole nitrogen source. The proteins were purified under denaturing conditions as described earlier [19] and was refolded using reported protocols [14]. Briefly, 250 µg of protein in 8 M urea was rapidly 20-fold diluted into the refolding mixture containing pre-chilled (4°C) solution of lipid or detergent in 20 mM Tris-HCl pH 9.5. The lipids and detergents used were: 50 mM DPC (*n*-dodecylphosphocholine), 30 mM DMPC (1,2 dimyristoyl-*sn*-glycero-3-phosphocholine), 150 mM 60PC (6∶0 diether PC; 1,2-di-*O*-hexyl-*sn*-glycero-3-phsophocholine) or 100 mM LDAO. After rapid dilution, OmpX refolding was achieved using one of two methods. (a) Heat shock was administered to the refolding reaction, at 70°C for 3 min, followed by incubation at 4°C for 15 min [14]. This led to rapid folding of OmpX. (b) The refolding reaction was incubated at 40°C for 3 h, which led to slow refolding of OmpX [14]. All refolding reactions were checked for completion by SDS-PAGE, using gel mobility shift of unboiled samples [14], [18], [19]. Quantification of refolding reactions was achieved by densitometry analysis using MultiGauge v2.3, and the fraction folded in each lane was calculated.

Excess urea in the refolded sample was removed by a quick dialysis step by carrying out one round of buffer exchange using a 1 kDa cut-off Vivaspin concentrator (GE Healthcare). This was followed by extensive centrifugation of the refolding reaction to remove trace amounts of precipitated or aggregated protein, using reported methods [42]. Unboiled samples were checked on SDS-PAGE for presence of any unfolded protein, after which the concentrations were adjusted as required for each experiment. Unless otherwise specified, the refolded samples were appropriately diluted to 30 µM protein (0.5 µg/µl) in 10 mM LDAO and 20 mM Tris-HCl pH 9.5 containing trace amounts (∼16 mM) of urea, for all spectroscopic measurements. Protein concentrations were estimated using A_280_ and a molar extinction coefficient of 34840 M^−1^ cm^−1^ (calculated using ProtParam tool [43]) was used. Samples for NMR and DSC experiments were prepared using similar protocols, but contained higher protein amounts required for these measurements. In these cases, the lipid concentration was also accordingly adjusted to maintain the LPR (lipid-to-protein ratio) at ∼330∶1. It must be noted that by ‘lipid’, when we refer to LDAO or DPC or 60PC, these molecules, particularly LDAO, are generally classified also as detergents, and they form micellar structures. DMPC, on the other hand, is a lipid and forms vesicles.

### Steady State Fluorescence and Circular Dichroism Measurements

Steady state fluorescence measurements were carried out on a Fluoromax-4 spectrofluorometer from Horiba Jobin-Yvon, France. Sample concentrations were adjusted to 15 µM (0.25 µg/µl) protein in 20 mM Tris-HCl pH 9.5 buffer containing 5 mM LDAO. All measurements were carried out at 25°C. Samples were excited at 295 nm and emission spectra collected between 310–400 nm, with a slit width of 2.5 nm for excitation and 3.0 nm for emission. Data were corrected for dark counts and buffer subtracted. Spectra were processed using OriginPro v8.0 and plots generated using SigmaPlot v11.0 (Systat Software).

Far-UV CD spectra were recorded on a JASCO J815 CD spectropolarimeter (JASCO Inc., Japan) equipped with Peltier temperature controller, using a 1 mm quartz cuvette, between 205–260 nm, using identical protein and lipid concentrations as for fluorescence. Near-UV CD was recorded between 230–340 nm, using five-fold higher protein and lipid concentrations of 150 µM (2.5 µg/µl) and 50 mM LDAO, respectively, in 20 mM Tris-HCl pH 9.5, using a 10 mm quartz cuvette. Free tryptophan was also recorded in the same buffer condition. Scan speeds were set to 100 nm/min with a 1 nm band width and data integration time of 1 s. Spectra were averaged over 3 accumulations, blank subtracted and smoothened. Secondary structure content was estimated using Reed’s reference [44], available with JASCO Spectra Manager v2.0. Plots were generated using SigmaPlot v11.0 (Systat Software).

### Lifetime Measurements

Time correlated single photon counting (TCSPC) was used to monitor fluorescence lifetimes of tryptophan residues, on a FluoroLog spectrofluorometer (Horiba Jobin-Yvon, France). Samples were excited at 295 nm and data were collected at 340 nm and 355 nm for the folded and unfolded protein samples, respectively. Instrument response function (IRF) determined using skimmed milk was found to be ∼827 ps. IRF deconvolution was carried out on all data and the decay curves were fit to a three exponential decay function using DAS6 v6.4 software (Horiba Scientific, France), to obtain α_i_ (amplitude fraction) and τ_i_ (corresponding lifetime). The average lifetime was then calculated using Στ = α_i_τ_i_ [45].

### NMR Data Acquisition

^1^H-^15^N HSQC (heteronuclear single quantum coherence) measurement of uniformly ^15^N labeled OmpX^M^ (0.16 mM) refolded in 20 mM Tris-HCl pH 9.5 containing 150 mM 60PC, was recorded on a Bruker AVANCE 500 spectrometer (Bruker BioSpin, GmbH) at 30°C, using similar conditions reported earlier [19], using standard pulse sequences available in the Bruker library. OmpX^M^ (0.13 mM) in 20 mM Tris-HCl pH 9.5 containing 100 mM LDAO, was recorded on a Bruker AVANCE III 700 MHz spectrometer (Bruker BioSpin, GmbH) at 40°C, using similar conditions as reported previously [14]. Data were processed using NMRPipe [46] and the plots were generated using Sparky [47].

### Equilibrium Unfolding Studies

Chemical denaturation was achieved using a GdmHCl (guanidine hydrochloride) gradient from 0 M to 6 M and the process was monitored through tryptophan fluorescence. Fluorescence measurements were carried out on a SpectraMax M5 microplate reader (Molecular Devices, USA) using 96-well flat bottom plates (Corning Inc., USA). A 200 µl reaction containing 10 mM LDAO with 0.5 µg/µl protein was incubated at 37°C, and progress of the reaction was monitored for 48 h. Equilibrium was achieved in 24 h. Spectra were recorded using λ_ex_ = 295 nm and emission range of 320–380 nm and step size of 1 nm. All spectra were subtracted using the respective blanks. Thermodynamic parameters were derived using fits of data derived from 2–3 independent experiments, to a two-state equation. Details are provided in the ‘Data Analysis’ section.

### Anisotropy Measurements

Steady state anisotropy measurements were recorded on Fluoromax-4 (Horiba Jobin-Yvon, France). Samples subjected to equilibrium unfolding for 24 h (as described above), were excited at 295 nm and anisotropy was measured at a fixed λ_em_ of 340 nm, which corresponded to the λ_em-max_ of the folded protein in the absence of any denaturant. A slit width of 5 nm was used for both excitation and emission wavelengths, with data integration time of 5 s, and 5 trials were acquired with a <2% standard error. Fits were carried out as described below.

### Data Analysis

Unless otherwise stated, all data were analyzed and plots were generated using SigmaPlot v11.0 (Systat Software). Total fluorescence intensity obtained from chemical denaturation experiments were converted to the unfolded fraction (*f_U_*) using the formula [48]:
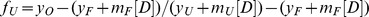



Here, *y_O_*, is the observed total fluorescence at a GdmHCl concentration *[D]*, *y_F_* and *m_F_* as well as *y_U_* and *m_U_* are the intercepts and slopes of the pre- and post-transition baselines. The folded fraction (*f_F_*) is given as *f_F_* = 1-*f_U_*. This data was directly fit to the two-state equation [48]–[50]:
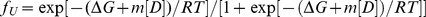
where, Δ*G* represents the unfolding free energy in water (ΔG^0,H2O^_U_) with *m* representing the *m* value, and *R* and *T* are the gas constant and experiment temperature (in K), respectively. Thermodynamic parameters thus obtained were compared with values obtained by the linear extrapolation of ΔG^0^_U_ at various GdmHCl concentrations [50]. The slope and *y*-intercept corresponded to the *m* value and ΔG^0,H2O^_U_. This was used to obtain the C_m_ using, C_m_ = |ΔG^0,H2O^_U_/*m*|. C_m_ was also derived independently from fits to the two-state equation. Both methods provided comparable thermodynamic parameters. Furthermore, the anisotropy data for OmpX^M^ was fit to the three-state equation reported by Moon and Fleming [51].

### Thermal Denaturation Experiments

Protein unfolding and recovery with temperature was monitored using CD. Temperature was Peltier controlled and maintained within ±0.1°C of set values. Thermal melting and recovery were monitored between 4→95°C and 95→4°C, respectively, with a ramp rate of 1.0°C/min, and data collected at every temperature increment using a single-wavelength monitoring at 215 nm. All spectra were converted to molar ellipticities, as previously described [14], and normalized to the value obtained at 4°C. Data were averaged over 2–3 independent experiments. Near-UV CD wavelength scans were acquired at steps of 5°C, between 230–340 nm from 5°C→95°C. All spectra were blank subtracted and smoothened.

Thermal denaturation by microcalorimetry was carried out on a MicroCal VP-DSC System (GE Healthcare Lifesciences, Uppsala, Sweden), using ∼0.15 mM protein in 20 mM Tris-HCl pH 9.5 containing 50 mM LDAO. Higher protein concentrations were required for these measurements; nonetheless the LPR was maintained at 330∶1, to resemble the other experimental conditions. Data were recorded at a scan speed of 1°C/min and buffer subtracted data were first normalized for protein concentration, and then fit to an inbuilt two-state function using Microcal® Origin v7 (OriginLab Corporation), to obtain T_m_ and the calorimetric enthalpy ΔH.

## Results and Discussion

### Met Mutation does not Affect Barrel Refolding Efficiency or Protein Structure

OmpX^HN^ has three internal Met residues, at positions 18, 21 and 118, in addition to the ‘initiator’ Met. We have referred to the OmpX H100N mutant as OmpX^HN^ and the H100N:M18L:M21L:M118L quadruple mutant as OmpX^M^. In places where we refer to both the proteins, we have used the term OmpX. As described earlier, Figure 1A maps the position of the Met residues on the OmpX^HN^ crystal structure to the loop regions. Of the three Met residues, one, M118, is located in the periplasmic region, whereas M18 and M21 are situated in the extracellular region. We have previously reported that OmpX^HN^ can be rapidly refolded in various lipids and detergents using heat shock [14]. In this method, denatured OmpX, rapidly diluted in the refolding mix at 4°C, is subjected to a quick ‘heat shock’ at 70°C, which results in rapid protein folding and generation of a highly stable sample. Moreover, we have also observed that OmpX^HN^ can be refolded using the ‘slow folding’ method [14]. In this case, rapid dilution of urea-denatured OmpX in the refolding mixture is followed by incubating the reaction at 40°C for 3 h.

In order to test the effect of M→L mutation on the folding efficiency of OmpX^M^, we refolded both proteins using both heat shock and slow folding method, in various lipids and detergents, and compared the folding efficiency using band mobility shift [14], [19] on SDS-PAGE gels of unboiled samples. Figure 1B illustrates the resultant refolding efficiencies, obtained from the slow folding method, of samples that were not centrifuged prior to SDS-PAGE analysis. Densitometry analysis of the gel mobility shifts indicates that Met mutation does not cause any significant effect on protein folding. Additionally, total fluorescence measurements of tryptophan residues located at positions 76 and 140 for protein refolded in LDAO micelles are largely similar, and indicate no change in the local indole environment in either protein (Figure 1C). Circular dichroism (CD) measurements were also carried out and secondary structure content was estimated for both proteins. The results, summarized in Figure 1C and Table 1, indicate no difference between OmpX^HN^ and OmpX^M^ in terms of their secondary structure content.

**Table 1:** Secondary structure estimation from far-UV CD spectra of OmpX^HN^ and OmpX^M^.

Secondarystructure (%)	OmpX^HN,^ a	OmpX^M,^ a	OmpX^HN,^ b	OmpX^M,^ b
Helix	0.0	0.0	0.0	0.0
Beta sheet	44.3	43.6	47.6	48.8
Turn	10.3	11.3	9.8	8.8
Random	45.3	45.1	42.6	42.4
Total	100	100	100	100
RMS value	5.08	6.49	4.29	3.46

a Refolded by heat shock;

b refolded by slow refolding method. All values were calculated using Reed’s reference [44].

**Figure 2:**
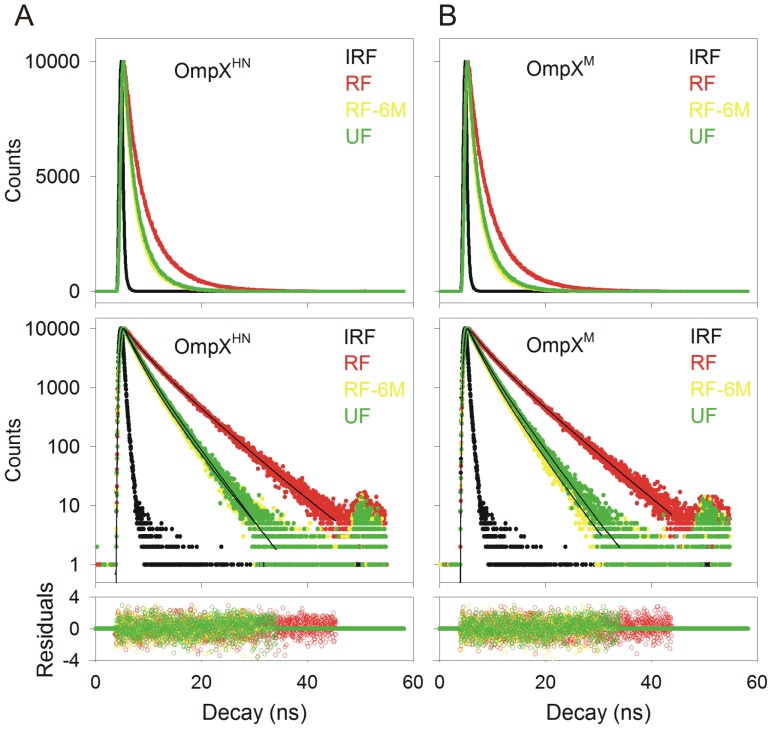
Fluorescence lifetime measurements to assess the local tryptophan environment. The upper panels show representative fluorescence decay profiles of the heat shock refolded protein (RF) for OmpX^HN^ (A) and OmpX^M^ (B). The refolded samples were unfolded by incubation in 6 M GdmHCl for 24 h, and the lifetime measurements so obtained are marked as RF-6M. Protein powder, directly dissolved in 8 M GdmHCl and containing no lipid or detergent, was used as control (UF). IRF refers to instrument response function, which was determined using skimmed milk. The middle panels show fits of the decay profiles to a three exponential function. Residuals obtained from the fit are in the bottom panel. Labels are color coded to match their respective spectra.

We carried out time resolved fluorescence measurements of the tryptophan residues, since lifetimes are highly sensitive to the local environment of the indole ring, and are excellent indicators of minor local variations in the barrel-lipid packing efficiency. Our data, summarized in Figure 2 and Table 2, fit well to a three state exponential function, similar to the decay profiles of tryptophan in soluble proteins. We obtained similar lifetime values for both OmpX^HN^ and OmpX^M^ in LDAO, suggesting that the local Trp environments for both systems are similar. This observation provides further support that both proteins have a comparable refolding efficiency, despite the mutations.

**Table 2:** Summary of observed tryptophan fluorescence lifetimes and amplitudes for OmpX^HN^ and OmpX^M^.

Protein	Refolding method	GdmHCl (M)	Lifetimes			Amplitudes			<τ>^c^	?^2,^ c
			τ_1_	τ_2_	τ_3_	a_1_	a_2_	a_3_		
OmpX^HN^	HS^a^	0	2.64	0.17	5.63	0.26	0.42	0.29	2.42	1.03
		6	1.90	1.85	3.70	0.56	0.32	0.16	1.32	1.08
	40^b^	0	2.79	2.93	5.87	0.33	0.35	0.30	2.83	1.07
		6	1.82	2.22	3.54	0.32	0.47	0.20	1.42	1.11
OmpX^M^	HS^a^	0	2.75	0.23	5.73	0.31	0.36	0.32	2.80	1.04
		6	1.82	1.99	3.45	0.30	0.49	0.19	1.32	1.07
	40^b^	0	2.87	2.89	5.93	0.34	0.36	0.29	2.82	0.99
		6	1.73	1.79	3.45	0.28	0.511	0.20	1.28	1.05
OmpX^HN^	Unfolded	8	1.91	2.11	3.60	0.29	0.45	0.24	1.55	1.10
OmpX^M^		8	1.83	2.15	3.57	0.29	0.43	0.26	1.59	1.04

a Refolding by heat shock at 70°C for 3 min;

b Refolding at 40°C for 3 h (slow folding);

c Average lifetime <τ> and goodness of fit to a three exponential decay function (χ^2^).

**Figure 3:**
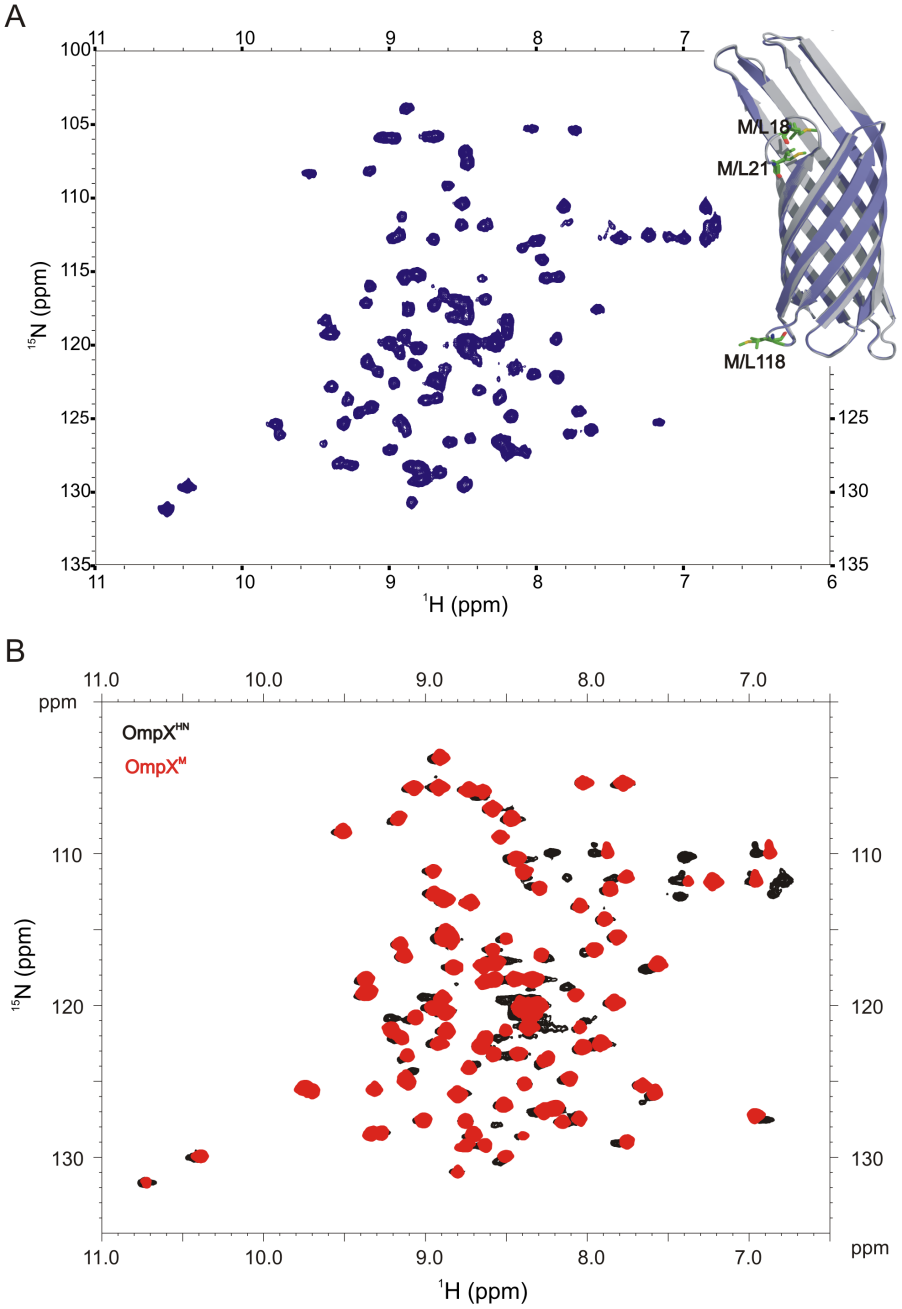
^1^H-^15^N HSQC spectra of uniformly ^15^N labeled OmpX^M^. (A) Spectrum of OmpX^M^ refolded in 150 mM 60PC acquired on a 500 MHz spectrometer using conditions reported earlier [19]. Inset illustrates the ribbon diagrams of OmpX^HN^ (grey) and OmpX^M^ (slate) with the methionines of OmpX^HN^ and the corresponding leucines of OmpX^M^ highlighted. The OmpX^M^ structure was generated by homology modeling using I-TASSER [64], with Met residues at positions18, 21, 118 replaced by Leu residues, and 1QJ8 [18] as the starting template. (B) Overlay of the ^1^H-^1^5N HSQC spectra of OmpX^HN^ (black) and OmpX^M^ (red), highlighting similar resonance distribution between both the proteins. The OmpX^HN^ spectrum is adapted from [14].

Furthermore, we recorded the heteronuclear ^1^H-^15^N HSQC spectrum of OmpX^M^ in 150 mM 60PC (6∶0 diether PC) in 20 mM Tris-HCl pH 9.5, using previously reported conditions for OmpX^HN^ [19], [20]. Structural variations occurring at the level of the backbone can readily be detected using HSQC, in addition to providing information on the folded state of the protein. In particular, modification of the hydrogen bonding pattern of the barrel or deviations in the local structure of the protein can be mapped readily using this method. This is possible because variations in the strength and nature of hydrogen bond formed by each residue would influence the observed chemical shift of the amide resonance in both the proton and nitrogen dimensions. Amides that form weak hydrogen bonds in β-sheet structures, or exist in a random coil conformation, experience an upfield shift in the resonance in both the dimensions of an HSQC spectrum. Internal hydrogen bond formation (within the protein) of the amide, of an amino acid adopting extended backbone φ-ψ values, results in a pronounced downfield shifted peak in the HSQC. The observed ^1^H-^15^N HSQC spectrum of OmpX^M^ in 60PC micelles is shown in Figure 3A. The spectrum is characterized by well-dispersed resonances, indicating that OmpX^M^ adopts a well-folded barrel in 60PC micelles. The spectrum compares well with previously reported spectra of OmpX^HN^ in both 60PC and LDAO micelles [14], [19], [20], indicating that Met mutations do not alter the overall structure of the protein. Furthermore, an overlay of the HSQC spectra of OmpX^HN^ and OmpX^M^ in LDAO is shown in Figure 3B. Nearly superimposable resonances for both proteins indicate that the Met→Leu substitution does not cause substantial changes in the barrel scaffold of OmpX.

### M→L Substitution Lowers Unfolding Free Energy of the Barrel by Destabilization

Most conserved substitutions are not destabilizing in proteins, since local structural rearrangements compensate well for any mutation-induced local destabilization. Indeed, it has been shown earlier that quadruple mutations involving insertion of Trp residues in OmpA, are well tolerated by the barrel [50]. Our data on OmpX^M^ also indicate no detectable difference from the parent OmpX^HN^. We probed this further by measuring the response of both proteins to equilibrium unfolding experiments. Both urea and guanidine hydrochloride (GdmHCl) serve as excellent denaturants to study unfolding kinetics. However, GdmHCl is considered a superior denaturant compared to urea, for transmembrane barrels, as demonstrated in the case of *E. coli* PagP [52]. We therefore examined the unfolding pathway of OmpX^HN^ and OmpX^M^ using GdmHCl, and determined the thermodynamic properties of the protein.

**Figure 4:**
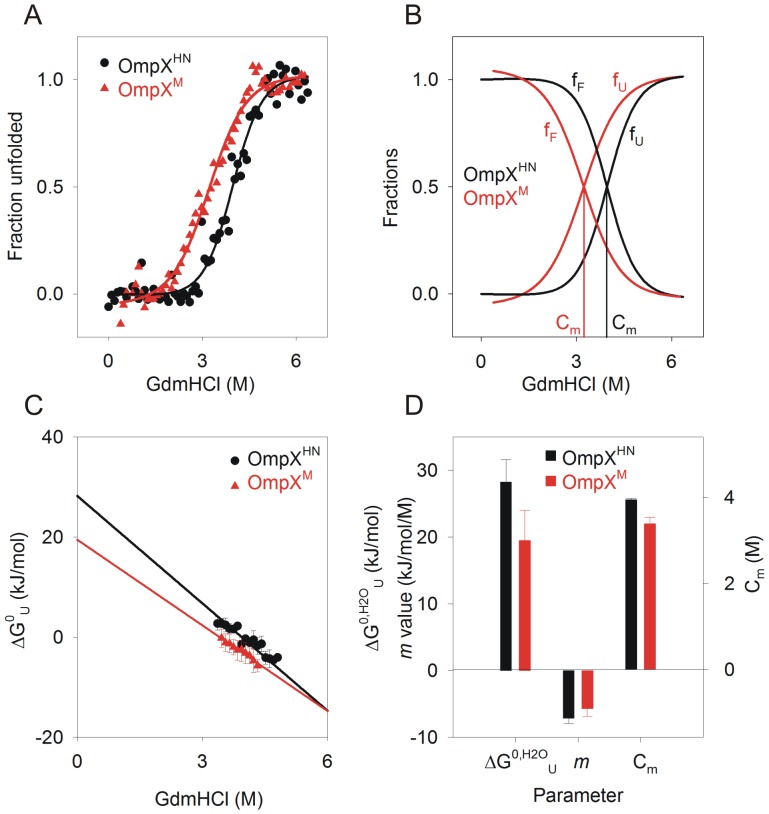
Equilibrium unfolding studies of heat shock refolded protein using chemical denaturation. (A) GdmHCl-induced denaturation curves of OmpX^HN^ (black circles) and OmpX^M^ (red triangles), were monitored using fluorescence, and unfolding transitions were fitted to a two-state equation to obtain C_m_. Error bars are omitted for purposes of clarity. The fits are shown as solid lines. (B) Folded (f_F_) and unfolded (f_U_) fractions are plotted against GdmHCl, to illustrate the change in C_m_ from ∼3.9 M to ∼3.3 M, for OmpX^HN^ (black) and OmpX^M^ (red). (C) Unfolding free energy at various GdmHCl concentrations were linearly extrapolated to obtain ΔG^0^_U_ in water. (D) Thermodynamic parameters (ΔG^0,H2O^_U_ in water, *m* value and C_m_) derived for OmpX^HN^ and OmpX^M^, using linear extrapolation.

Tryptophan fluorescence (W76 and W140) was monitored to measure the isothermal equilibrium unfolding of heat shock refolded OmpX, in 10 mM LDAO and an LPR (lipid-to-protein ratio; here lipid refers to LDAO, which is a detergent and forms a micellar assembly) of ∼330∶1, at 37°C. We were able to achieve unfolding equilibrium in ∼24 h, and the data points between 24 h and 48 h were comparable. We therefore used the 24 h datasets for all our thermodynamic analyses. Figures 4A and 4B demonstrate representative unfolding and refolding transients for both proteins. The equilibrium curves fit well to a two-state equation (Figure 4A), indicating that both proteins undergo a cooperative unfolding process from the folded state to the GdmHCl-denatured state. Additionally, no unfolding intermediates are observed in these experiments. A lowering of unfolding cooperativity is noticeable in OmpX^M^, wherein the unfolding transition occurs between a wider GdmHCl concentration compared to that of OmpX^HN^. We derived unfolding free energies in water (ΔG^0,H2O^_U_) for both proteins as described earlier [49], [50], which are in the range of ∼28.2 kJ/mol and ∼19.5 kJ/mol for OmpX^HN^ and OmpX^M^, respectively. When compared with reported unfolding free energies for other 8-stranded barrels, the values obtained for OmpX are poorer, suggesting that the barrel stability for OmpX is lowered in micellar systems, as opposed to that in lipid vesicles. This observation is in contrast to the reported behavior of OmpA (another homologous 8-stranded barrel from *E. coli*) in octyl maltoside micelles [11], indicating that homologues may show different stability based on the strength of protein-lipid interactions [8]. It must also be noted that the denaturing effect of urea, as compared to guanidine, on experiments for free energy estimations especially in detergent systems can influence the calculated values.

Our equilibrium unfolding experiments provided us with a change in the unfolding free energy (ΔΔG^0^_M→L_) of ∼8.5 kJ/mol for the conserved substitution of the three methionine residues of OmpX^HN^ to leucines. This lowering of free energy indicates that upon Met mutation, the OmpX barrel is considerably less stable compared to the parent protein. This difference could arise due to several factors, the key contributing agents involve protein refolding efficiency, change in the accessible surface arising from decreased interaction affinity between protein and lipid, and lowered barrel stability. The gel mobility shifts and spectroscopic measurements (Figure 1) indicate no detectable change between the refolding efficiency of both proteins. Therefore, we examined the *m* values, which, for water-soluble proteins, correlate with change in solvent accessible surface area (ASA) as the protein transitions from the folded to unfolded form [52]. It has been shown that some of the barrels studied so far have *m* values in the range of ∼18 kJ/mol^.^M (OmpW), ∼23 kJ/mol^.^M (PagP) and ∼37 kJ/mol^.^M (OmpLA) [52]. These values were derived from a detailed study of barrel unfolding in large unilamellar vesicles of DLPC (1,2-dilauroyl-*sn*-glycero-3-phosphocholine), under conditions with negligible lateral pressure from hydrophobic mismatch or curvature stress. Our experiments provide *m* values (∼6–7 kJ/mol^.^M) that are towards the lower end of the ASA values observed earlier for soluble and membrane proteins [52]. Such reduced *m* values could arise from the micellar environment used in this study, which may allow easy solvent access to the protein and is affected by the presence of denaturants. Comparison of the calculated *m* values between OmpX^HN^ and OmpX^M^ indicates no significant variations between the two proteins. This suggests that the exposed and solvent accessible surface areas of both proteins upon refolding are fairly similar.

We further explored the source of destabilization by comparing the thermodynamic parameters calculated for OmpX^HN^ and OmpX^M^ (Figure 4D) refolded using the heat shock method [14]. The change in unfolding free energies correlates well with the reduction in C_m_ values (the chemical denaturation midpoint) for OmpX^M^. This indicates that Met substitutions cause destabilization of the barrel. This is also evident from changes in the pre-transition baseline for OmpX^M^, which indicates local destabilization of the barrel well before the unfolding mid-point is achieved (Figures 4A and 4B). To further understand the origin of this destabilization, we refolded the protein using the ‘slow refolding’ approach by rapidly diluting urea-denatured OmpX into the refolding mixture and incubating the reaction at 40°C for 3 h. Care was taken to ensure that the final buffer conditions, LPR and dilution factors were similar to the samples prepared by heat shock.

**Figure 5:**
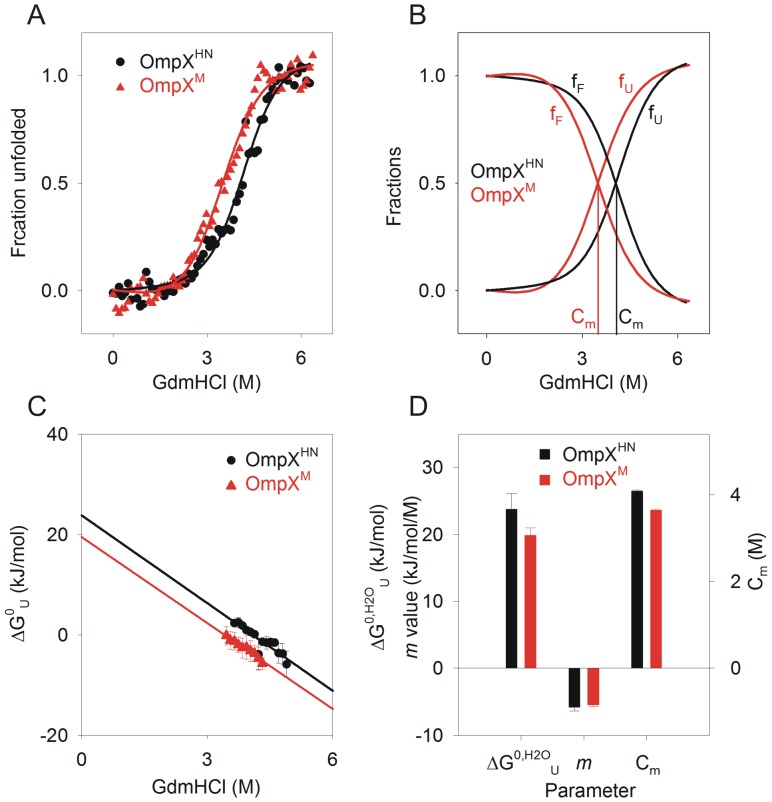
GdmHCl-induced equilibrium unfolding of OmpX^HN^ and OmpX^M^ refolded by the slow folding method. (A) GdmHCl-induced denaturation was monitored using Trp fluorescence, and the unfolding transitions of OmpX^HN^ (black circles) and OmpX^M^ (red triangles) were fit to a two-state equation to obtain C_m_. Error bars are omitted for purposes of clarity. The fits are shown as solid lines. (B) Folded (f_F_) and unfolded (f_U_) fractions, plotted against GdmHCl, indicate a C_m_ of ∼4.0 M and ∼3.6 M for OmpX^HN^ (black) and OmpX^M^ (red), respectively. Unfolding free energy (C) at various GdmHCl concentrations were linearly extrapolated to obtain ΔG^0,H2O^_U_ in water. Thermodynamic parameters (ΔG^0,H2O^_U_ in water, *m* value and C_m_) derived from the equilibrium unfolding experiments are plotted in (D).

Thermodynamic data obtained from equilibrium unfolding experiments of refolded protein samples generated by the slow folding method, are summarized in Figure 5. As in the case of heat shock refolded OmpX^M^, protein samples prepared by the slow folding method lack a proper baseline for the initial guanidine concentrations. It is believed that in such cases, there is a substantial population of unfolded protein even in the absence of a denaturant, which may interfere in estimations of equilibrium thermodynamic parameters [53]. However, in the case of OmpX^M^, SDS-PAGE and spectroscopic measurements (see Figure 1) provide evidence for near-complete refolding for this protein. Based on this, we are tempted to speculate that the absence of a distinct baseline and the corresponding lowered cooperativity of unfolding, which is evident in the case of OmpX^M^ (Figure 5A), is due to the existence of a lipid-adsorbed species in the refolded sample. These species, also referred to as lipid-associated species, are those protein molecules that transiently associate with the lipid or detergent. Based on the extent of this association, they may exhibit various levels of folding and may also lead to aggregation. However, we do not observe any aggregation in OmpX even upon prolonged storage [14]. We believe that OmpX^M^ therefore exists in equilibrium between refolded and adsorbed states, both of which are stable under ambient conditions; the latter, however, readily undergoes unfolding in the presence of denaturants, giving rise to the observed loss in cooperativity of unfolding.

The calculated values for ΔG^0,H2O^_U_ for these samples are ∼23.7 kJ/mol and ∼19.8 kJ/mol for OmpX^HN^ and OmpX^M^, respectively. These results are in good agreement with the values obtained from the heat shock refolded protein, indicating that the sample generation process by itself does not influence the estimates. Notwithstanding the lowered ΔG^0,H2O^_U_ for OmpX^HN^ when compared with similar proteins of the β-barrel category, this value is further reduced due to Met replacement. The observed ΔΔG^0^_M→L_ of ∼8.5 kJ/mol for the heat shock refolded protein and ∼4.0 kJ/mol for samples generated by the slow folding method cannot be ignored. Remarkably, these mutations are at the loop regions of OmpX^HN^, a protein which has documented evidence of substantial tolerance to loop modifications and barrel tailoring [22], [23]. In an attempt to understand the unfolding event in greater detail, we probed the process using fluorescence anisotropy.

### Unfolding Intermediate for OmpX^M^ and Strong Protein-lipid Interactions for OmpX^HN^ Observed from Fluorescence Anisotropy

Steady state fluorescence measurements of unfolding and refolding kinetics in proteins are most widely employed for the determination of protein thermodynamic and kinetic parameters. While this method reliably provides extensive information in several soluble protein complexes as well as the membrane protein family, it must be noted that the latter are studied in a lipid environment. One can envision that the unfolding and refolding processes can be populated with intermediate states that are bound to various amounts of lipid. Additionally, lipid molecules may preferentially bind local hydrophobic pockets of the partially folded protein. Such regions are often enriched with aromatic amino acids. Indeed, a recent study on kinetic analysis of OmpA discusses the implications of surfactant- and lipid-bound intermediates on the inferred unfolding and refolding pathways [11]. Furthermore, the occurrence of hysteresis in thermodynamic studies of several membrane proteins introduces complications in data interpretation [54], since multi-state processes can appear to be an apparent two-state event [52].

Our steady state fluorescence data suggest a two-state unfolding process for both proteins. Fluorescence anisotropy measurement can help in supplementing the information obtained from Trp emission spectra. Anisotropy measures the extent of depolarization of the emitted fluorescence, which is determined by the degree of rotational freedom allowed for the fluorophore under various conditions. In the folded protein which is embedded in lipid micelles, Trp residues are located near the solvent-lipid interface, and have restricted motion. Hence, the anisotropy values are higher, and reflect the rotation of the bulk protein-lipid entity. As the protein unfolds and loses affinity to the lipid molecules, anisotropy values are expected to be lowered. Therefore, anisotropy measurements can capture possible unfolding and refolding intermediates that have bound lipid at the indole vicinity, which otherwise exhibit cooperative two-state profiles in steady state measurements.

**Figure 6:**
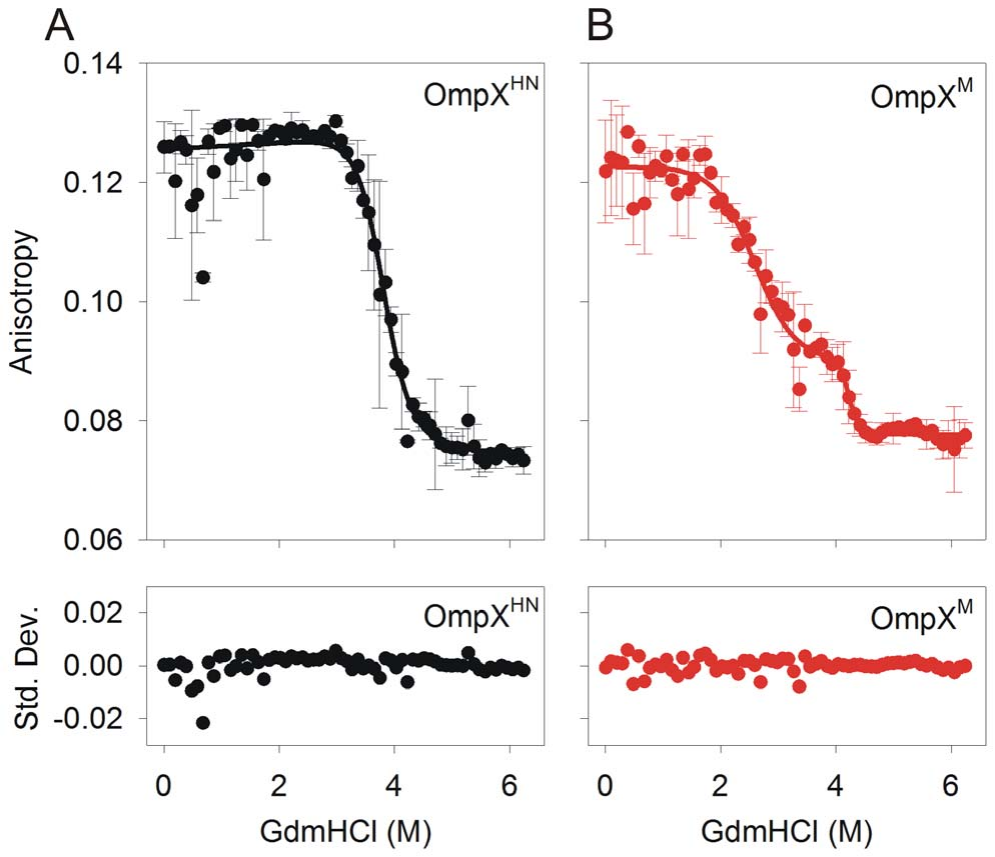
Steady state Trp fluorescence anisotropy measurements. OmpX^HN^ (A) and OmpX^M^ (B) refolded using heat shock in 10 mM LDAO were subjected to GdmHCl denaturation for 24 h and anisotropy values were recorded. Fits to a two-state equation for OmpX^HN^ and a three-state equation for OmpX^M^, are shown as solid lines. Note that the unfolding of OmpX^M^ follows a three state transition, while OmpX^HN^ fits well to a two-state, as indicated by the residuals (shown below each curve as standard deviation (Std. Dev.)).

OmpX^HN^ shows a cooperative transition from the folded to the unfolded state, with increasing GdmHCl, when monitored using fluorescence anisotropy (Figure 6A). A fit to a two-state equation yielded the C_m_ to be ∼3.9 M, which matches the C_m_ obtained from the fluorescence unfolding measurements. When we compare OmpX^HN^ data of Figure 4A with that of Figure 6A, the difference in cooperativity of unfolding becomes evident from both experimental results. Fluorescence emission measurements indicate that the unfolding event is less cooperative than the anisotropy data, due to the marginal reduction in fluorescence intensity between 0.5–3.0 M (Figure 4A), while there is no corresponding change in the anisotropy in this GdmHCl range. This data suggests that the buried indole rings of OmpX^HN^ undergo solvent exposure in lower GdmHCl concentrations in the range of 0.5–3.0 M, leading to reduction in fluorescence intensity at λ_em-max_, due to non-radiative relaxation of the excited state. However, the rings are still retained in the rigid lipid environment, accounting for the high anisotropy values we observe. These changes are, however, marginal in OmpX^HN^. Surprisingly, the variation in unfolding cooperativity is more dramatic in OmpX^M^.

Interestingly, we observe a three-state unfolding for OmpX^M^ (Figure 6B), with the first unfolding transition providing a C_m1_ of ∼2.5 M, which is in close agreement with the previous calculations from equilibrium unfolding monitored using fluorescence. An additional transition is observed between 4.0–5.0 M GdmHCl, with a C_m2_ of ∼4.1 M. We are tempted to conclude that the transient unfolding intermediate observed at ∼3–4 M GdmHCl possibly relates to a lipid-bound form of OmpX^M^. A comparison of the progress in unfolding monitored by fluorescence and anisotropy in OmpX^M^ indicates analogous rates from both experiments (OmpX^M^ data from Figures 4A and Figure 6B), unlike OmpX^HN^. Furthermore, the unfolding transition of OmpX^HN^, monitored using anisotropy, occurs between ∼3.2–4.8 M, which accounts for an ∼1.6 M GdmHCl range. On the contrary, OmpX^M^ unfolding occurs over a wider range of the denaturant, between ∼1.8–4.6 M, providing a transition zone of ∼2.8 M GdmHCl for the unfolding process. The latter is nearly ∼1.5-fold wider than OmpX^HN^, which in turn reflects a poorer cooperativity in unfolding for OmpX^M^.

Based on these observations, we conclude that there exists stabilizing contacts between OmpX^HN^ and LDAO micelles, which accounts for the high(er) unfolding free energy for this protein. The loss in unfolding cooperativity upon conversion of OmpX^HN^→OmpX^M^ is evident in both the fluorescence emission spectra and anisotropy experiments, suggesting that Met mutations affect the global barrel stability. A recent report on simulation studies of barrel unfolding indicate a sequential process for the unfolding event, with protein-lipid contacts mediating the stabilization of intermediate states [55]. In OmpX^HN^, destabilization of protein-lipid interaction requires a threshold denaturant concentration; when this is reached, the unfolding event is nucleated. On the other hand, in OmpX^M^, the regions harboring the mutation may act as multiple local destabilization points, leading to a more rapid and less cooperative unfolding process. Once the OmpX^M^ barrel is sufficiently unfolded from these regions, the core segment persists at the intermediate GdmHCl concentrations. This intermediate of OmpX^M^ is likely to resemble the unfolding intermediate of OmpX^HN^. Beyond 4 M GdmHCl, the protein core rapidly loses affinity to LDAO in both OmpX^HN^ and OmpX^M^, and undergoes complete unfolding.

### Susceptibility of Slow-refolded OmpX to Thermal Denaturation is Altered upon M→L Substitution

**Figure 7:**
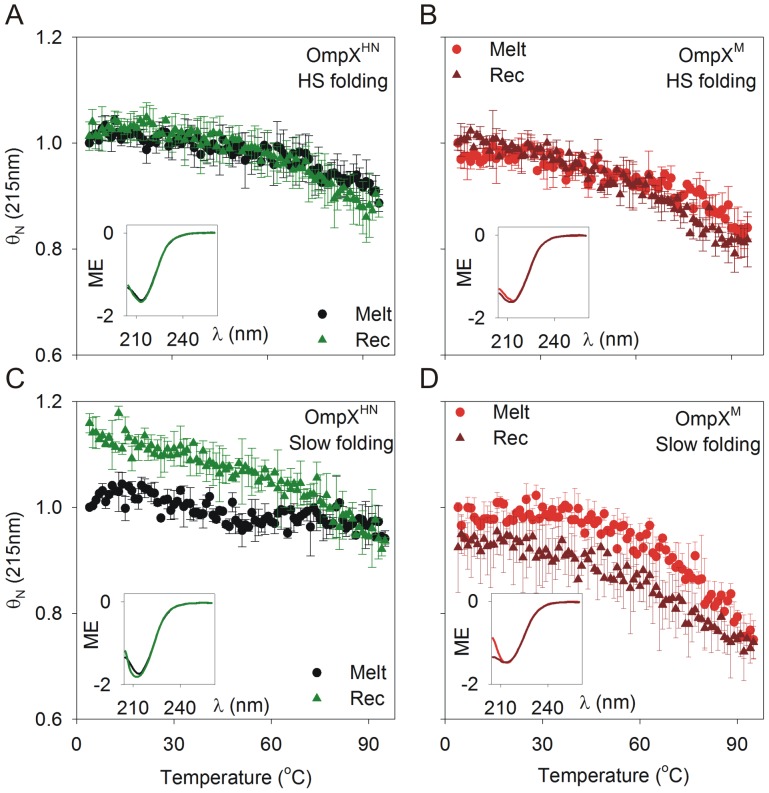
Thermal denaturation (melting) and recovery of OmpX^HN^ and OmpX^M^ monitored using far-UV CD. Melting (Melt; circle) and recovery (Rec; triangle) of OmpX^HN^ (A) and OmpX^M^ (B), refolded by heat shock (HS folding) in 10 mM LDAO, from 4→95°C and 95→4°C, respectively. (C) and (D) represent data for thermal denaturation and recovery for OmpX^HN^ and OmpX^M^ samples generated by slow folding at 40°C for 3 h (slow folding). All insets represent far-UV CD scans of samples before and after thermal melts. Labels are color coded to match their respective spectra. ME (molar ellipticity) values are in units of deg cm^2^ dmol^−1^ and are factored by 10^6^.

Protein-lipid interaction efficiency can also be monitored using heat as a denaturant. In membrane proteins, thermal denaturation, more often than not, results in irreversible unfolding and protein aggregation, when the protein-lipid interactions are altered [56]. We have previously demonstrated that OmpX^HN^ undergoes reversible thermal denaturation in LDAO micelles and despite being of mesophilic origin, the protein shows a T_m_ (mid-point of thermal denaturation) of ∼107°C [14]. We therefore compared the extent of unfolding of OmpX^M^ using circular dichroism (CD) and microcalorimetry (DSC). The protein concentrations required for CD and DSC are different; hence, in order to retain the same LPR used in chemical denaturation experiments, CD studies were carried out in 10 mM LDAO (far-UV CD) or 50 mM LDAO (near-UV CD), and 50 mM LDAO was used for DSC measurements. Figure 7 illustrates the results obtained for thermal denaturation and recovery measurements using CD, for both OmpX^HN^ and OmpX^M^, refolded using both heat shock and slow refolding. The data were obtained by monitoring CD values for the unfolding and recovery experiments at 215 nm, which corresponds to the negative maximum obtained from the n-π* transition for β-sheets. Our experimental set-up prevents data acquisition beyond 100°C. As noted earlier, OmpX does not undergo complete unfolding at 95°C, precluding us from obtaining proper baselines for the completely unfolded species [14]. We therefore compared the normalized molar ellipticity values for both proteins.

Both OmpX^HN^ and OmpX^M^ prepared using the heat shock method show nearly superimposable unfolding and recovery transitions, with negligible hysteresis at higher temperature (Figure 7A and 7B). Closer examination of the data indicates that OmpX^M^ undergoes marginally greater unfolding compared to OmpX^HN^. This is exemplified further in the samples prepared by slow folding at 40°C (Figure 7C and 7D). We observe an ∼20% increase in the folded population after thermal denaturation and recovery for OmpX^HN^. This could possibly arise because the slow folding method may generate an undetectable population of adsorbed protein species or barrels with locally misfolded regions and therefore undergo different levels of compaction. Subjecting these proteins to thermal denaturation provides the system with the necessary energy that allows them to overcome such unfavorable local minima, leading to formation of the native form, during the refolding (recovery) pathway of the experiment. OmpX^M^, on the contrary, shows a small, yet significant difference in the recovered protein population. We did not observe detectable amounts of unfolded protein, from SDS-PAGE analysis and light scattering experiments (which monitor the presence of soluble aggregates at ∼320–330 nm). We therefore attribute this loss in the secondary structure content of OmpX^M^ to its poor(er) interaction efficiency with lipid micelles, which is aggravated upon thermal denaturation.

**Figure 8:**
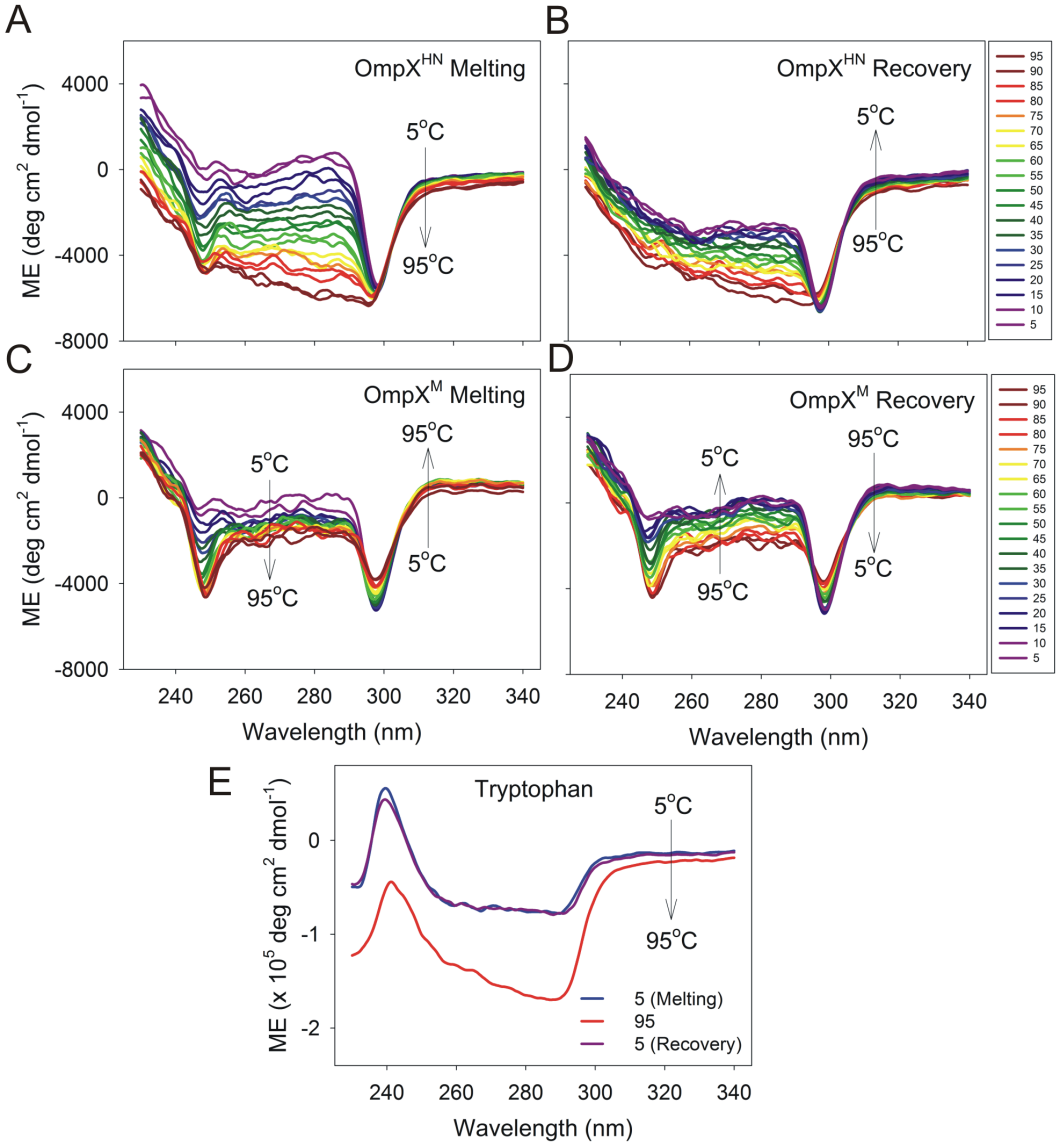
Near-UV CD monitoring spectral changes of heat shock refolded protein with temperature. OmpX^HN^ and OmpX^M^ exhibit weak near-UV CD spectra that undergo minor variations during thermal denaturation and recovery measurements. Thermal denaturation (A and C) and recovery (B and D) of OmpX^HN^ (A and B) and OmpX^M^ (C and D) are colored based on the temperature gradient, and the direction in which the change occurs is marked in each spectrum. Also shown is the observed spectrum of free tryptophan in 50 mM LDAO (E).

We also examined the presence of a tertiary fold in both the proteins, using near-UV CD, and monitored the change of this spectrum with temperature. In globular proteins, tertiary CD is a result of aromatic interactions in the hydrophobic core and is characterized by weak signals in the 260–290 nm region. However, in the case of β-barrel membrane proteins, aromatic residues are distributed at the barrel girdle and face the lipid milieu. These residues anchor the protein to the lipid environment and do not usually form the kind of aromatic clusters seen in soluble proteins. Hence, the near-UV CD profile of OmpX is different, and is characterized by two weak signals at ∼245 nm and ∼295 nm (Figure 8). Furthermore, the spectrum shows an unusual response to a rise in temperature, wherein we observe an increase in the signal upon heating. We speculate that what we observe is a consequence of the interaction of plane polarized light with Trp residues in the OmpX-micelle complex, and may not directly correspond to the protein’s tertiary structure. This is further supported by the observed thermal denaturation spectra of free tryptophan in LDAO micelles (Figure 8E), which gives rise to two signals at ∼240 nm and ∼290 nm. While it is tempting to speculate that the observed differences between the spectra of OmpX^HN^ and OmpX^M^ are due to the different strengths of interaction of either protein with LDAO, direct evidence for such interactions from the CD spectra is presently unavailable.

**Figure 9:**
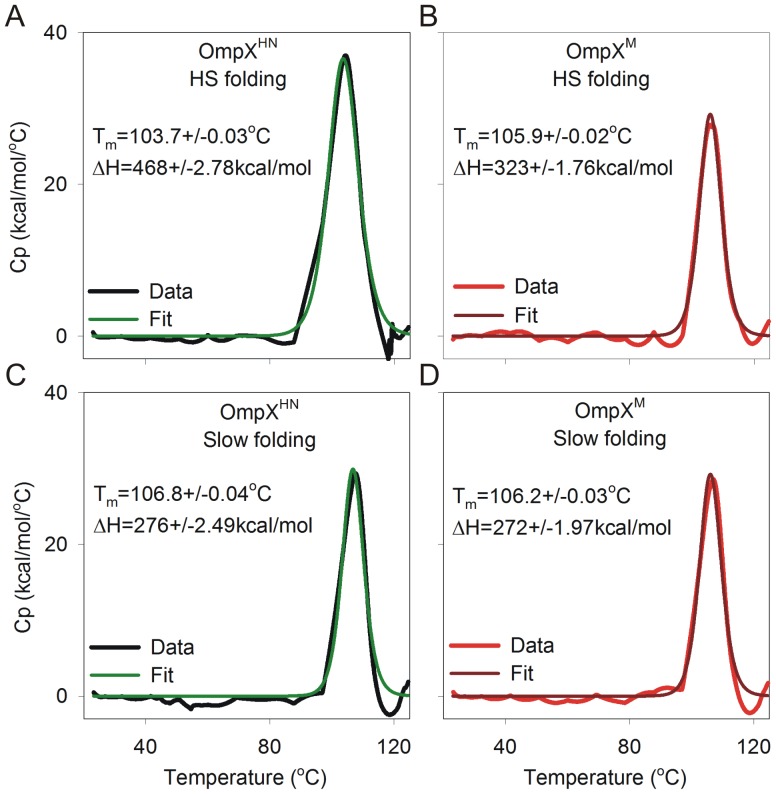
Differential scanning calorimetric analysis. DSC data for OmpX^HN^ (A and C; black and green) and OmpX^M^ (B and D; red and brown), generated using heat shock folding (A and B) or slow refolding (C and D) methods. Unfolding T_m_ and ΔH derived from fits to a two-state model, are indicated beside each curve.

Results of DSC measurements of heat shock refolded OmpX^HN^ and OmpX^M^ are shown in Figure 9A and 9B, and those from slow folding in Figure 9C and 9D. In line with our previous observations [14], all samples show T_m_ values between 103–107°C. The observed change in free energy (ΔG^0,37^_U_) from DSC measurements, calculated at 37°C, provides estimations that are several fold higher than the ΔG^0,H2O^_U_ values obtained from the chemical denaturation. This stems partly from the observed thermal stability of the protein-micellar complex to thermal denaturants. However, this primarily from large errors in estimation of the ΔC_p_ values for these systems due improperly defined pre- and post-transition baselines corresponding to the folded and unfolded proteins, respectively. This is because the unfolding of membrane proteins is accompanied by both exothermic and endothermic events, resulting in inaccurate values for change in specific heat capacity [56]. Hence, direct interpretation of the free energy values obtained from DSC measurements of OmpX is not possible. The major difference between the two proteins refolded by heat shock stems from the variation in the enthalpy values, which may reflect differing stabilities of both proteins. However, we observe that both OmpX^HN^ and OmpX^M^, prepared by the slow folding method, show comparable enthalpy and free energy values, which are lower than their heat shock refolded counterparts. These results are complementary to the CD thermal denaturation measurements of OmpX prepared by slow folding, wherein an increase in secondary structure content is observed for OmpX^HN^ (Figure 7C), while OmpX^M^ undergoes an ∼20% loss in structure upon heating (Figure 7D). Since CD primarily monitors changes in the protein secondary structure content, it may serve as a straightforward tool for analysis of membrane proteins without interference from heat capacity changes associated with the transitions of empty micelles. We observe higher order oligomers for both proteins in our refolding reactions, which could also interfere in the unambiguous determination of the thermodynamic parameters from DSC measurements.

We are tempted to conclude that the differences in ΔH values, of heat shock refolded OmpX^HN^ from OmpX^M^ or both the samples generated by slow folding, originates from the level of hydration of refolded barrel samples. In soluble proteins, the observed enthalpy and heat capacity changes are due to disruption of interactions within the protein and with solvent molecules [57], [58]. In membrane proteins, however, the protein molecule is in dynamic equilibrium between intramolecular (hydrophobic, electrostatic, van der Waals), protein-solvent, protein-lipid and lipid-lipid interactions [56]. The overall differences in thermodynamic parameters are influenced by the number of lipid molecules surrounding the protein [59]; therefore, the lipid (or detergent) plays a significant role in determining the ΔH of the system. Furthermore, it is speculated that membrane proteins do not undergo complete unfolding and may exist in lipid-bound forms even upon denaturation [59]. In our studies, the observed increase in ΔH for OmpX^HN^ (heat shock refolded) indicates that the protein undergoes transition from a well-folded state that is associated with fewer water molecules to an unfolded form. On the other hand, OmpX^M^ is marginally destabilized due to the mutation and has a greater exposed protein surface and poorer protein-lipid interactions, thereby leading to the observed lowering of enthalpy, upon thermal denaturation. Furthermore, samples generated by the slow folding method show lower enthalpy values due to the inefficiency of this process in generation of stably refolded protein, compared to the heat shock method. However, owing to the complexity in studies of membrane proteins with DSC, it is presently difficult to derive concrete conclusions from DSC measurements.

## Conclusion

Membrane proteins demonstrate an acute dependence for folding and stability on the refolding environment, lipid chain length and head group, among other factors [8], [9], [42], [60]. Previous studies on transmembrane β-barrels of bacterial origin have revealed a general destabilization and slower folding kinetics upon mutation of residues in the transmembrane region and aromatic girdle [10]. However, mutations of the extra-membrane loop region are expected to show greater ramification on barrel function, and in line with previous reports, not affect barrel stability [22]. By contrast, our studies reveal that conserved substitution of methionine residues present in the loop region of OmpX, to leucines, adversely affects the equilibrium free energy of the system. Furthermore, our observation of folding intermediates during the folding process of OmpX^M^ indicates that a universal folding pathway, involving the formation of an adsorbed species, is adopted by most β-barrels during folding. We believe that while this intermediate is short-lived in OmpX^HN^, the mutation allows us to detect this species, due to changes in the interaction efficiency of the protein with its lipid or detergent environment. Similar studies carried out on point mutations of loop segments of the human mitochondrial voltage dependent anion channel-2 (hVDAC-2), a 19-stranded transmembrane barrel, also support the important role played by loop residues on barrel stability [61].

The lowering of cooperativity in our equilibrium unfolding experiments, upon mutation of methionine residues, is noteworthy. The Met→Leu substitution could result in the formation of the observed refolding intermediates, which in turn lowers the unfolding free energy. We are able to capture the intermediate only in our anisotropy measurements, whereas direct analysis of Trp fluorescence does not allow us to observe this intermediate. We speculate that the destabilization could arise from local weakening of protein-lipid interactions at the interface, creating an ‘Achilles’ heel’, which, in turn, allows easy access of the barrel-lipid interior, to denaturants. It is plausible that the marginal increase in hydrophobicity of leucine is sufficient to cause this destabilization. Conserved Met→Leu substitutions even in loop segments of membrane proteins may result in altered biophysical properties of the system and affect protein-lipid interactions. Hence, the effect of conserved substitutions in membrane proteins should not be underestimated, as it may cause an adverse effect on the protein’s behavior. Furthermore, it is tempting to speculate that modulation of protein-lipid interactions in membrane proteins may bear a subtle, yet significant influence on the structure and thermodynamic properties of these molecules and may serve as key contributors of membrane protein recycling. Significant differences in the thermodynamic properties of β-barrels adopting similar secondary and tertiary structural scaffolds could therefore arise from subtle variations in the primary sequence.
